# Evaluation of Biochemical and Epigenetic Measures of Peripheral Brain-Derived Neurotrophic Factor (BDNF) as a Biomarker in Huntington’s Disease Patients

**DOI:** 10.3389/fnmol.2019.00335

**Published:** 2020-01-23

**Authors:** Ashley Gutierrez, Jody Corey-Bloom, Elizabeth A. Thomas, Paula Desplats

**Affiliations:** ^1^Department of Neuroscience, School of Medicine, University of California, San Diego, San Diego, CA, United States; ^2^Department of Neuroscience, The Scripps Research Institute, La Jolla, CA, United States; ^3^Department of Pathology, School of Medicine, University of California, San Diego, San Diego, CA, United States

**Keywords:** Huntington’s disease, brain-derived neurotrophic factor, DNA methylation, saliva, blood, biomarker, epigenetics, clinical symptoms

## Abstract

Huntington’s disease (HD) is an autosomal-dominant neurodegenerative movement disorder that presents with prominent cognitive and psychiatric dysfunction. Brain-derived neurotrophic factor (BDNF) plays an important role in the pathophysiology of HD, as well as other neurodegenerative and psychiatric disorders, and epigenetic alterations in the complex BDNF promoter have been associated with its deregulation in pathological conditions. BDNF has gained increased attention as a potential biomarker of disease; but currently, the conflicting results from measurements of BDNF in different biofluids difficult the assessment of its utility as a biomarker for HD. Here, we measured BDNF protein levels in plasma (*n* = 85) and saliva (*n* = 81) samples from premanifest and manifest HD patients and normal controls using ELISA assays. We further examined DNA methylation levels of *BDNF* promoter IV using DNA derived from whole blood of HD patients and healthy controls (*n* = 40) using pyrosequencing. BDNF protein levels were not significantly different in plasma samples across diagnostic groups. Plasma BDNF was significantly correlated with age in control subjects but not in HD patients, nor were significant gender effects observed. Similar to plasma, salivary BDNF was correlated with age only in control subjects, with no gender effects observed. Importantly, we detected significantly lower levels of salivary BDNF in premanifest and manifest HD patients compared to control subjects, with lower BDNF levels being observed in premanifest patients within a predicted 10 years to disease onset. Salivary and plasma BDNF levels were not significantly correlated with one another, suggesting different origins. DNA methylation at four out of the 12 CpG sites studied in promoter IV were significantly altered in HD patients in comparison to controls. Interestingly, methylation at three of these CpG sites was inversely correlated to the Hospital Anxiety and Depression Scale (HADS) scores. *BDNF* promoter methylation was not correlated with motor or cognitive scores in HD patients, and was not associated with sex or age in neither disease nor control groups. Conclusion: Our studies show that BDNF protein levels are decreased in saliva; and *BDNF* promoter methylation increased in blood in HD subjects when compared to controls. These findings suggest that salivary BDNF measures may represent an early marker of disease onset and DNA methylation at the *BDNF* promoter IV, could represent a biomarker of psychiatric symptoms in HD patients.

## Introduction

Huntington’s disease is an inherited, progressive neurodegenerative disorder characterized by chorea, movement dysfunction, cognitive impairment, and behavioral disturbances (The Huntington’s Disease Collaborative Research Group, 1993). The prevalence rate of HD in the United States is approximately 5 per 100,000 people (Lanska, 2000; Ho et al., 2001). HD is caused by a CAG trinucleotide repeat expansion in the first exon of the *HTT* gene encoding the HTT (The Huntington’s Disease Collaborative Research Group, 1993). Because the gene mutation is known, diagnostic and predictive genetic testing is available for HD; however, biomarkers for HD are still greatly needed to predict disease onset, to assess the diversity and severity of symptoms and to monitor disease progression (Ross et al., 2014). Furthermore, HD biomarkers could serve for monitoring clinical trials of disease-modifying therapeutics (Ross et al., 2014).

A major limitation in the development of biomarkers for CNS disorders is the inaccessibility of brain tissue; therefore, screening of peripheral tissues that may serve as proxy for brain degeneration is crucial. Cerebral spinal fluid (CSF) is thought to represent the biofluid most similar to the brain environment, however, CSF collection is an invasive technique that requires a lumbar puncture, which can be painful and lead to side effects and complications (Evans, 1998). With regards to peripheral sources, investigations in blood have dominated the field for decades, however, other peripheral fluids for biomarker studies have included saliva, urine, and sweat.

One candidate biomarker for HD is BDNF, which plays an important role in survival, growth and differentiation of neurons (Lu, 2003; Greenberg et al., 2009). Decreased levels of BDNF, especially in the striatum, are thought to play a crucial role in HD pathogenesis. A loss of normal huntingtin-mediated *BDNF* gene transcription, and dysregulation of BDNF expression by the mutant form of huntingtin, both contribute to the reduced levels of BDNF in the brain, which has been observed in post-mortem brain tissue from human HD patients (Ferrer et al., 2000; Zuccato et al., 2001), as well as in several transgenic mouse models of HD (Zuccato et al., 2001; Duan et al., 2003; Zhang et al., 2003). Notably, several studies have shown that overexpression of BDNF to the brain ameliorates HD phenotypes in the R6/1 and R6/2 mouse models (Zuccato et al., 2001, 2008; Gharami et al., 2008; Xie et al., 2010), and, in one study, BDNF overexpression *in vivo* was found to rescue HD phenotypes in YAC128 mice (Xie et al., 2010). Moreover, BDNF knock-out mice exhibit many symptoms reminiscent of HD transgenic mice (Canals et al., 2004). These studies suggest therapeutic implications for elevating BDNF levels in this disease.

In addition to widespread expression throughout the CNS (Ma et al., 2012), BDNF is also present in blood (Yamamoto and Gurney, 1990; Radka et al., 1996; Hohlfeld et al., 2006), and studies have shown that BDNF can cross the blood-brain barrier (Pan et al., 1998), suggesting that peripheral measures of BDNF have relevance to brain function and vice versa. However, conflicting literature exists on the levels of BDNF in blood of HD patients, with one study reporting decreased levels of BDNF transcripts in whole blood of HD patients (Krzyszton-Russjan et al., 2013); another showing increased levels of BDNF protein in platelets (Betti et al., 2018) and a third study showing no significant changes in BDNF protein levels in plasma or serum in HD patients (Zuccato et al., 2011) in comparison to healthy control subjects. Here we sought to further investigate blood measures of BDNF in HD patients, as well as exploring BDNF levels in another peripheral fluid, saliva.

With regards to markers of *BDNF* gene activity, analyses have focused on DNA methylation of the *BDNF* promoter in various neurological disorders. Specifically, studies have demonstrated changes in DNA methylation at the BDNF promoter in blood samples from patients with Alzheimer’s disease (AD) (Rao et al., 2012; Xie et al., 2017), major depression (Januar et al., 2015; Kang et al., 2015) and schizophrenia (Ikegame et al., 2013). Such studies have suggested that *BDNF* promoter methylation might be a useful peripheral biomarker of disease symptoms observed in these disorders. Based on these findings, we further examined DNA methylation levels at *BDNF* promoter IV in whole blood samples from HD patients and tested for any associations between the *BDNF* DNA methylation levels and clinical presentation in HD patients.

## Materials and Methods

### Participants

This study was approved by the University of California, San Diego Institutional Review Board in accordance with the requirements of the Code of Federal Regulations on the Protection of Human Subjects. Informed consent from all subjects was obtained prior to their participation. Patients were recruited from the University of California, San Diego (UCSD) HD Clinical Research Center. HD patient criteria included a definitive diagnosis of HD with family history and an expanded trinucleotide CAG repeat of 40 or more. Normal controls had no reported history of neurological or psychiatric disorders, and no use of psychoactive substances or medications. Demographic information was collected at the time of saliva collection, including sex, age, and CAG repeat length. This information is provided in Table 1. Patients were assessed for cognitive and motor function using the MMSE (score range 0–30) (Gluhm et al., 2013), the Total Motor Score from the UHDRS (score range 0–124), and TFC (range 0–13). Patients were assessed for anxiety and depression symptoms using the HADS. The predicted age to onset of disease symptoms was calculated using the HD conditional onset probability calculator, according to Langbehn, and using the default probability to onset of 0.6^1^ (Langbehn et al., 2010).

**TABLE 1:** Characterization of cases included in this study.

	**HD**	**PM**	**NC**
**Plasma**			
N	21	30	33
Female:Male	13:8	17:13	17:16
Age (years) ± SD	56.8 ± 11.1	41.2 ± 12.9	50.9 ± 14.6
CAG repeat	43.1 ± 3.07	42.0 ± 2.93	NA
**Saliva**			
N	20	23	38
Female:Male	11:9	10/13	22/16
Age (years) ± SD	54.5 ± 12.7	43.6 ± 14.5	48.1 ± 14.2
CAG repeat	43.9 ± 2.91	42.3 ± 11.2	NA
**DNA methylation**			
N	10	11	18
Female:Male	6:4	6/5	12/6
Age (years) ± SD	58.2 ± 12.5	41.8 ± 10.6	55.2 ± 22.3
CAG repeat	43.5 ± 2.67	43.6 ± 2.87	NA

*HD, Huntington’s disease; PM, Pre-manifest; NC, Normal controls; NA, Not applicable.*

### Saliva Collection

All donors were asked to refrain from smoking, eating, drinking, or oral hygiene procedures for at least 1 h prior to samples collection, and then rinsed their mouth thoroughly with water 15–20 min prior to sample collection. Saliva samples were collected between 10:00 am and 4 pm. using the passive drool method according to previously established protocols (Granger et al., 2012). Roughly two to three milliliters of unstimulated whole saliva were obtained. Samples were immediately frozen at −20°C at the time of collection, then stored at −80°C. At the time of use, saliva samples were thawed and centrifuged at 10,000 *g* for 10 min at 4°C to remove insoluble material and cellular debris. Supernatants were collected and used for all assays.

### Plasma Collection

Blood was drawn by venipuncture into 2 ml lavender/EDTA tubes. EDTA/whole blood was mixed well by inversion and spun at 900 × *g* for 15 min. The top plasma layer was transferred into 4 × 1 ml aliquots and snap frozen and stored at −80°C.

### BDNF Measurements

Brain derived neurotrophic factor levels in plasma were measured using a commercially available kit according to our previous studies (Corey-Bloom et al., 2017). BDNF levels in saliva were measured using a sandwich ELISA modified from a protocol optimized for salivary BDNF (Mandel et al., 2011). A 96-well microtiter plate was incubated overnight at 4°C with a monoclonal mouse anti-human BDNF (100 ul; clone 35928; Calbiochem, San Diego, CA, United States), diluted to 1 μg/ml in filter-sterilized PBS, pH 7.4. The plate was manually washed three times with TBS + 3% tween (TBST), allowing the plate to soak for 1 min each time. Wells were blocked with 300 μl of 3% BSA in PBST for 2.5 h at room temperature. Standards ranging from 15.6 to 500 pg/ml were prepared fresh for each assay using a full-length, homodimeric recombinant BDNF (rBDNF; Peprotech, Rocky Hill, NJ, United States). Following blocking, the plate was washed five times and 50 ul of sample (diluted 1:1 in blocking solution), was added to the wells and assayed in triplicate. The plate was incubated for 2.5 h at RT with agitation and then washed five times. Subsequently, a polyclonal chicken anti-human BDNF (100 ul; 2.5 μg/ml; Promega) was added to the plate and incubated for 2.5 h. The plate was washed five times with PBST, then anti-chicken IgY-HRP (1 μg/ml; Promega) was added to each well for a 1-h incubation at RT. After the final wash, TMB (tetramethylbenzidine; Promega) substrate was added to each well, followed by quenching with 1M HCl and quantified by reading at 450 nm. The amount of BDNF in each sample was calculated using the regression equation from the standard curve for the plasma samples. For the saliva samples, the amount of BDNF is expressed as a relative amount based on absorbance. All assays were performed by operators blinded to the clinical state of the participant.

### Quantification of DNA Methylation at BDNF Promoter

We interrogated 12 CpG sites at promoter IV of the *BDNF* gene by pyrosequencing (performed by EpigenDx) using the probe ADS221-FS2re that covers the region chr11:27723246–27723191 and includes six CpG sites; and the probe Ads3858-FS mapped to chr11:27723144–27723076 and including another six CpG sites. Briefly, DNA was extracted from 1 mL of whole blood from *n* = 18 control subjects and *n* = 21 HD cases using QIAamp DNA Blood Midi Kit (Qiagen) as per manufacturer’s protocol. DNA (500 ng) was bisulfite-converted with the EZ DNA Methylation−Gold^®^ Kit (Zymo Research, CA, United States). Bisulfite−treated DNA (25 ng) was amplified using HotStarTaq DNA Polymerase (Qiagen, CA, United States) and the following standard PCR conditions. One primer was biotin-labeled and HPLC purified in order to purify the final PCR product using sepharose beads. PCR product was bound to Streptavidin Sepharose HP (GE Healthcare Life Sciences), after which the immobilized PCR products were purified, washed, denatured with a 0.2 μM NaOH solution, and rewashed using the Pyrosequencing Vacuum Prep Tool (Pyrosequencing, Qiagen), as per the manufacturer’s protocol. Next, 0.5 μM of sequencing primer was annealed to the purified single stranded PCR products. 10 μL of the PCR products were sequenced by Pyrosequencing on the PSQ96 HS System (Pyrosequencing, Qiagen) following the manufacturer’s instructions.

The methylation status of each CpG site was determined individually as an artificial C/T SNP using QCpG software (Pyrosequencing, Qiagen). The methylation level at each CpG site was calculated as the percentage of the methylated alleles divided by the sum of all methylated and unmethylated alleles. The mean methylation level was calculated using methylation levels of all measured CpG sites within the targeted region of each gene. Each experiment included non-CpG cytosines as internal controls to detect incomplete bisulfite conversion of the input DNA. In addition, a series of unmethylated and methylated DNA are included as controls in each PCR. Furthermore, PCR bias testing was performed by mixing unmethylated control DNA with *in vitro* methylated DNA at different ratios (0, 5, 10, 25, 50, 75, and 100%), followed by bisulfite modification, PCR, and Pyrosequencing analysis. The methylation level at each CpG site was calculated as the ratio of C (methylated cytosine) relative to T (unmethylated cytosine). Mean percent methylation was calculated as the average of individual CpGs.

### Statistics

All statistical analyses were performed using Graphpad software (Prism) or JASP Stats. The distribution of the data values in each diagnostic group was tested for normality using the Kolmogorov–Smirnov normality test. BDNF values in all groups showed a normal distribution. Outliers within each plasma and saliva diagnostic groups were determined using Iglewicz and Hoaglin’s test for multiple outliers using a *z* cutoff = 3.5. For the plasma data, this analysis resulted in removing one outlier in the HD group. For the saliva data, this analysis resulted in removing three outliers in the normal control group. Differences between diagnostic groups were determined using One-way ANOVA followed by Dunnett’s post-test comparing all groups to the normal control group or when comparing two groups, a Student’s *t* test was used. The effects of sex and age on the diagnostic differences in BDNF levels were determined by ANCOVA. Linear regression analysis (Spearman correlation) was used to compare BDNF levels against age and the clinical variables, which were not normally distributed. For clinical correlations, PM and HD patients were combined. *P*-values were adjusted for multiple test comparisons using a Bonferroni correction. Sex differences were determined using Student’s *t* test (unpaired; two-tailed). Statistical significance was defined by a *P* value of less than or equal to 0.05.

## Results

### Study Cohort

The samples [plasma (*n* = 85), saliva (*n* = 81), and whole blood (*n* = 39)] used in this study correspond to HD patients and healthy controls recruited from the University of California, San Diego (UCSD) HD Clinical Research Center over a period of 3 years. HD patient criteria included a definitive diagnosis of HD with family history and an expanded trinucleotide CAG repeat of 40 or more, giving them “gene (+)” status. Gene (+) patients consisted of both premanifest patients (PM), who were asymptomatic; and manifest, symptomatic HD patients (designated “HD”; Table 1). There were no significant differences in sex ratios or age between the HD and control groups. The patients providing plasma, saliva or whole blood samples were separate cohorts with only approximately 25% of subjects contributing more than one sample type.

### BDNF Levels in Plasma of HD Patients

**FIGURE 1:**
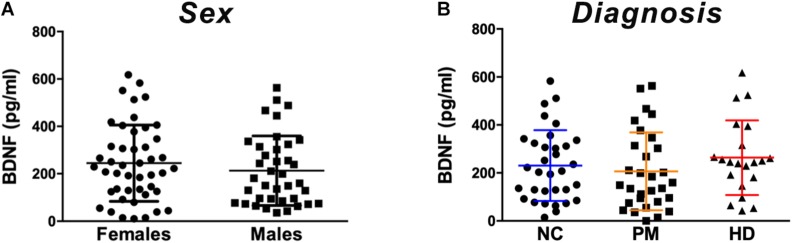
Plasma BDNF levels according to sex and diagnostic group. BDNF levels in plasma (50 ul) were determined by ELISA. **(A)** BDNF levels did not differ by sex among subjects included in our study as per Student’s *t* test, unpaired; two-tailed. **(B)** No significant differences were detected according to diagnostic group between premanifest (PM), manifest HD (HD), and normal controls (NC) as per One-way ANOVA. Data is shown as individual BDNF levels ± SD.

We quantified BDNF protein in plasma from HD patients using a commercially available BDNF ELISA kit (Promega). This kit showed excellent reproducibility in a subset of plasma samples that were tested approximately 1 year apart (*r* = 0.797; *p* = 0.0004; Supplementary Figure S1). We did not detect any sex differences in BDNF levels (*p* = 0.3468 unpaired *t*-test; Figure 1A). BDNF levels in plasma were positively correlated with age in control subjects (Pearson *r* = 0.500; *p* = 0.006; Table 2), although this association was absent in HD patients (Pearson *r* = 0.1945; *p* = 0.1714; Table 2).

**TABLE 2:** Correlations between plasma BDNF levels and clinical data in HD patients and control subjects.

	**Age**	**CAG**	**HADS**	**TFC**	**MMSE**	**MOTOR**
**HD**						
Pearson *r*	0.1945	−0.04949	0.0619	−0.1804	−0.09626	0.2247
95% confidence interval	−0.09404 to 0.4529	−0.3310 to 0.2401	−0.2677 to 0.3786	−0.4412 to 0.1085	−0.3722 to 0.1953	−0.06263 to 0.4777
*P* value (two−tailed)	0.1714	0.7329	0.7081	0.2053	0.5061	0.1129
*P* value summary	ns	ns	ns	ns	ns	ns
**NC**						
Pearson *r*	**0.500**	NA	−0.094	NA	NA	NA
95% confidence interval	0.1142 to 0.6920	NA	−0.5618 to 0.4235	NA	NA	NA
*P* value (two-tailed)	**0.006**	NA	0.736	NA	NA	NA
*P* value summary	**^∗∗^**	NA	ns	NA	NA	NA

*NC, normal controls; HD patients included premanifest (PM) and manifest HD patients; HADS, Hospital Anxiety and Depression Scale; TFC, Total Functional Capacity; MMSE, Mini-Mental State Examination; MOTOR, the Unified Huntington’s Disease Rating Scale Motor component. Bold lettering denotes significant p values. ^∗∗^*P* = 0.006.*

Comparing across diagnostic groups, we did not detect any significant differences in BDNF levels between PM nor HD patients in comparison to control subjects (*p* > 0.05, one-way ANOVA; Figure 1B). Plasma BDNF levels were not correlated with CAG repeat length nor with any clinical measure, including the MMSE, TFC, total motor score (MOTOR), and the HADS (Table 2).

### BDNF Levels in Saliva of HD Patients

**FIGURE 2:**
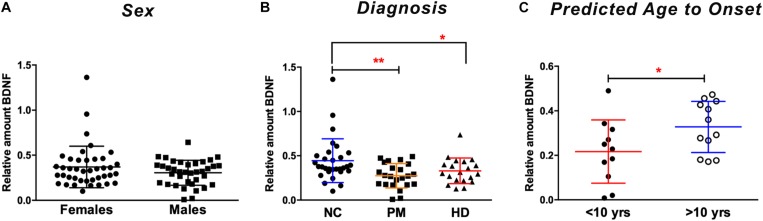
Salivary BDNF levels according to sex and diagnostic group. BDNF levels in saliva (50 ul) were determined by ELISA. **(A)** No significant differences in BDNF were observed by sex (Student’s *t* test, unpaired; two-tailed). **(B)** BDNF levels are lower in premanifest (PM) and manifest HD (HD) cases when compared to normal controls (NC). ^∗^*p* < 0.05; ^∗∗^*p* < 0.01 in comparison to NC group as per One-way ANOVA. **(C)** BDNF levels are significantly lower in subjects who are >10 years from their predicted age of onset compared to those who are <10 years from predicted onset. ^∗^*p* = 0.05. Predicted age to onset was calculated using the Langbehn formula (Langbehn et al., 2010). Data is shown as individual BDNF levels ± SD.

We next explored the use of saliva as an alternative peripheral non-invasive biofluid for BDNF measurements. We used a modified ELISA method optimized for saliva measurements in human saliva samples, as previously described (Mandel et al., 2011). We did not detect sex differences in the levels of BDNF in saliva (*p* = 0.7324 unpaired *t*-test; Figure 2A), but, similar to plasma, salivary BDNF was positively correlated with age in controls subjects (Pearson *r* = 0.387; *p* = 0.021; Table 3). However, neither age nor sex were significant covariates in the diagnostic comparisons of BDNF levels (ANCOVA, *p* = 0.212 and *p* = 0.328 for age and sex, respectively). Comparing salivary BDNF levels across diagnostic groups, we detected a significant decrease in salivary BDNF in both PM and HD patients compared to control subjects (*p* < 0.01 and *p* < 0.05 for PM and HD, respectively One-way ANOVA; Figure 2B). We next calculated the predicted age to onset of PM patients according to Langbehn et al. (2010) to determine if BDNF levels might be different in those patients closer to disease onset. We found a significant decrease in BDNF levels in those patients who had a predicted age to onset of <10 years, compared to those with a predicted onset of >10 years (Figure 2C).

**TABLE 3:** Correlations between salivary BDNF levels and clinical data.

**Saliva**						

	**Age**	**Age of Onset**	**HADS**	**TFC**	**MMSE**	**MOTOR**
**HD**						
Pearson *r*	−0.1857	−0.2189	−0.05931	−0.1844	−0.04182	0.1952
95% confidence interval	−0.4635 to 0.1254	−0.6031 to 0.2477	−0.3762 to 0.2700	−0.4798 to 0.1486	−0.3654 to 0.2908	−0.1376 to 0.4884
*P* value (two-tailed)	0.2391	0.3537	0.7273	0.2746	0.8087	0.2469
*P* value summary	ns	ns	ns	ns	ns	ns
**NC**						
Pearson *r*	**0.387**	NA	−0.210	NA	NA	NA
95% confidence interval	0.05432 to 0.5686	NA	−0.6066 to 0.2699	NA	NA	NA
*P* value (two-tailed)	**0.021**	NA	0.387	NA	NA	NA
*P* value summary	**^∗^**	NA	ns	NA	NA	NA

*NC, normal controls; HD patients included premanifest (PM) and manifest HD patients; HADS, Hospital Anxiety and Depression Scale; TFC, Total Functional Capacity; MMSE, Mini-Mental State Examination; MOTOR, the Unified Huntington’s Disease Rating Scale Motor component. Bold lettering denotes significant p values. ^∗^*P* = 0.021.*

As with the plasma BDNF data, we looked for possible correlations between salivary BDNF levels and clinical measures. Similar to what we observed in plasma, salivary BDNF levels in patients did not correlate with clinical scores including MMSE; TFC; total motor score or HADS score (Table 3).

A subgroup of subjects (*n* = 20) from all cohorts that provided a saliva sample for this study also provided a blood sample, hence we investigate the correlation between BDNF levels in plasma and saliva. We did not detect a significant correlation between plasma and salivary levels (Pearson *r* = −0.218; *p* = 0.435; data not shown), suggesting that the BDNF protein detected in the saliva is not originating nor transported from the blood.

### DNA Methylation Alterations at BDNF Promoter IV

**FIGURE 3:**
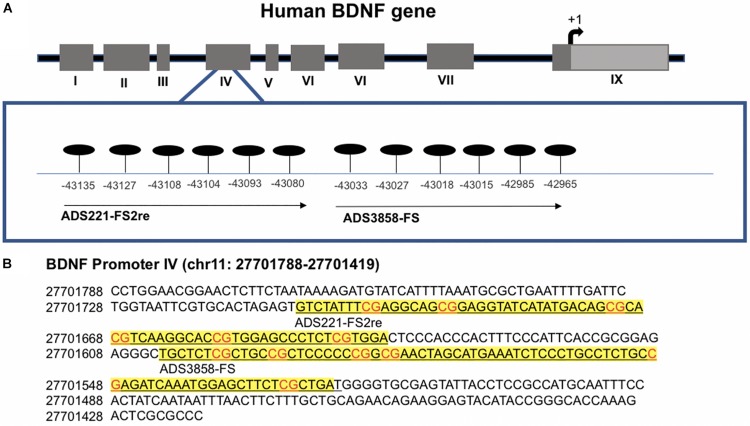
Methylation sites in the BDNF promoter IV region. **(A)** Schematic representation of human BDNF gene indicating alternative promoters. CpG sites analyzed are detailed in enlarged box. Position is in relation to transcription start site. **(B)** Detailed sequence of BDNF promoter IV. Underlined text in yellow boxes indicate regions analyzed with each probe by pyrosequencing. Genomic position is based on chromone build GRCh37.p13 primary assembly.

**FIGURE 4:**
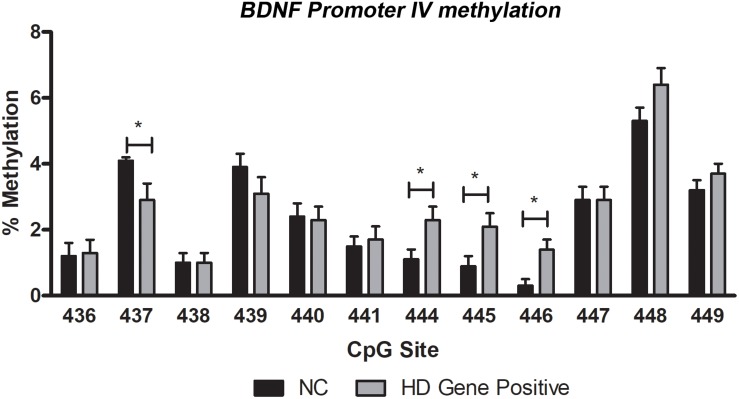
DNA methylation levels of BDNF promoter IV in blood. Methylation was quantified by pyrosequencing and expressed as mean percent methylation representing the averaged value of cases (HD Gene+) or control subjects (NC) for each of individual CpG. Data represents mean value ± SEM. ^∗^*p* < 0.05 as per two-tailed Student’s *t*-test.

As a measure of *BDNF* gene activity, we quantified methylation levels in DNA isolated from whole blood samples from *n* = 21 HD patients and *n* = 18 normal controls (Table 1) by pyrosequencing. For the initial analysis, both HD and PM cases were combined to increase sample size and statistical power. We focused on 12 CpG sites in promoter IV of the *BDNF* gene (Figure 3) as this region has been previously reported as differentially methylated in Alzheimer’s disease and other neurological conditions (Rao et al., 2012; Ikegame et al., 2013; Januar et al., 2015; Kang et al., 2015; Xie et al., 2017). The mean methylation levels for each CpG tested is shown in Table 4 for both HD patients and healthy control subjects. We found that four CpG sites were differentially methylated in HD patients, three of the sites showing increased methylation (CpG 444, CpG 445, and CpG 446) and one site, CpG 437, showing decreased methylation levels in comparison to control subjects (Table 4 and Figure 4). For these four CpG sites, we further separated the HD cases into different stages (PM, late pre-manifest (LPM) and manifest HD), and compared methylation levels across stages. We did not observe significant changes in methylation in the early stages of disease, however, progressive increases in methylation at sites 444, 445, and 446 were observed across all stages (Supplementary Figure S2). We did not detect a correlation between promoter IV DNA methylation levels to age nor an effect of gender, supporting that these epigenetic changes observed in HD patients are likely associated with the presence of the *HTT* mutation (Table 5 and Supplementary Figure S3).

**TABLE 4:** DNA methylation status at selected CpG sites from BDNF promoter IV.

**CpG site**	**Genomic position Chr11**	**Methylation (%)^1^**		***p*-value^2^**
		**NC**	**HD Gene (+)**	
436	27701529	1.2 (0.0 – 4.6)	1.3 (0.0 – 5.1)	0.89
437	27701549	4.1 (3.1 – 5.5)	2.9 (0.0 – 5.9)	0.032^∗^
438	27701579	1.0 (0.0 – 4.2)	1.0 (0.0 – 4.1)	0.849
439	27701582	3.9 (0.0 – 5.7)	3.1 (0.0 – 7.7)	0.196
440	27701591	2.4 (0.0 – 4.4)	2.3 (0.0 – 5.8)	0.889
441	27701597	1.5 (0.0 – 3.8)	1.7 (0.0 – 4.2)	0.706
444	27701644	1.1 (0.0 – 3.5)	2.3 (0.0 – 7.0)	0.028^∗^
445	27701657	0.9 (0.0 – 3.5)	2.1 (0.0 – 5.3)	0.023^∗^
446	27701668	0.3 (0.0 – 2.6)	1.4 (0.0 – 3.7)	0.002^∗∗^
447	27701672	2.9 (0.0 – 6.9)	2.9 (0.0 – 6.5)	0.961
448	27701691	5.3 (0.0 – 8.9)	6.4 (3.5 – 11.3)	0.079
449	27701699	3.2 (2.0 – 6.3)	3.7 (0.0 – 6.1)	0.312

*^1^Methylation levels are expressed as a mean percentage and (min–max) values. ^2^As per Student’s *t* test (unpaired; two-tailed). Significant differences are denoted by asterisks, ^∗^ and ^∗∗^, for the shown *p*-values.*

**TABLE 5:** Correlations between BDNF promoter IV methylation and clinical measures in HD patients.

	**Age**	**CAG**	**MMSE**	**MOTOR**	**TFC**	**HADS**
**Site 444**						
Pearson *r*	–0.1823	0.2772	–0.2358	–0.1612	0.1574	–0.5179
95% confidence interval	−0.5693to0.2708	−0.1756to0.6332	−0.6059to0.2182	−0.5544to0.2908	−0.2944to0.5517	−0.7761to−0.1109
*P* value (two-tailed)	0.4289	0.2239	0.3034	0.4852	0.4955	0.0162
*P* value summary	ns	ns	ns	ns	ns	^∗^
**Site 445**						
Pearson *r*	–0.1862	0.141	–0.1579	–0.179	0.1387	–0.533
95% confidence interval	−0.5720to0.2670	−0.3096to0.5399	−0.5520to0.2939	−0.5670to0.2739	−0.3117to0.5383	−0.7843to−0.1315
*P* value (two-tailed)	0.419	0.5421	0.4942	0.4374	0.5487	0.0128
*P* value summary	ns	ns	ns	ns	ns	^∗^
**Site 446**						
Pearson *r*	–0.09932	0.2046	–0.2974	–0.1395	0.1238	–0.5048
95% confidence interval	−0.5093to0.3474	−0.2492to0.5847	−0.6462to0.1542	−0.5388to0.3110	−0.3253to0.5274	−0.7690to−0.09335
*P* value (two-tailed)	0.6684	0.3737	0.1905	0.5465	0.5928	0.0196
*P* value summary	ns	ns	ns	ns	ns	^∗^
**Site 437**						
Pearson *r*	–0.2749	0.1826	–0.07029	–0.2762	0.1962	0.2656
95% confidence interval	−0.6317to0.1780	−0.2705to0.5695	−0.4873to0.3728	−0.6325to0.1766	−0.2574to0.5789	−0.2006to0.6337
*P* value (two-tailed)	0.2279	0.4283	0.7621	0.2255	0.394	0.2578
*P* value summary	ns	ns	ns	ns	ns	ns

*MMSE, Mini-Mental State Examination; the Unified Huntington’s Disease Rating Scale Motor component (Motor); TFC, Total Functional Capacity; *p*-values shown are corrected for multiple comparisons. ^∗^*p* < 0.05.*

We next tested whether methylation status at these four sites were correlated with BDNF levels in plasma or saliva. For subjects providing whole blood for the DNA methylation analysis, we were able to match subsets to plasma and saliva data (*n* = 20 and *n* = 16 for plasma and saliva, respectively), although these samples were not taken on the same day. We did not detect significant correlations between plasma BDNF and DNA methylation levels for any of the CpGs tested (Spearman *r* value range = −0.06 to 0.322; uncorrected *p*-value range = 0.12 to 0.99). In contrast, we observed a negative correlation between salivary BDNF levels and DNA methylation at site #444 (Spearman *r* = −0.489; *p* = 0.049) and #446 (Spearman *r* = −0.298; *p* = 0.044); although the *p*-values were no longer significant after a multiple test correction (*p* = 0.176 and *p* = 0.196 for site #444 and #446, respectively).

### Correlations Between BDNF Promoter IV Methylation and Clinical Data

The analysis of the correlation between DNA methylation levels at the four CpG sites significantly altered in in HD patients with clinical symptoms and disease data showed interesting results. Methylation levels at CpG sites 444, 445, and 456 were inversely correlated to anxiety and depression (Spearman *r* = -0454 to -0483; *p* < 0.05), as determined by the HADS, a widely used measure of anxiety and depression in patients (Table 5). In contrast, methylation at CpG site 437, which was decreased in HD patients, was not correlated to the HADS score (Table 5), suggesting that the increases in methylation at CpG sites 444, 445, and 456 might be especially relevant. We did not detect significant correlations between methylation levels and motor or cognitive symptoms, including MMSE, the UHDRS and the TFC (Table 5).

Control subjects also were tested for depression symptoms using the HADS index and surprisingly, there was no significant difference between HADS score in HD patients vs. controls (20.8 ± 4.3 vs. 16.6 ± 2.8; *p* = 0.43; Student’s *t* test; two-tailed; unpaired). Correlations between HADS scores and methylation at all 12 CpGs was assessed separately in control subjects and significant correlations were observed for methylation at CpG sites 436, 438, and 447, however, only the *p*-value for the correlation to site 436 survived multiple tests correction (Pearson *r* = −0.671; corrected *p*-value = 0.027; data not shown).

## Discussion

Multiple lines of evidence indicate that reduced BDNF expression plays a crucial role in HD pathogenesis (Zuccato et al., 2001, 2008; Gharami et al., 2008; Xie et al., 2010), suggesting that peripheral detection of BDNF levels and/or gene activity might represent a relevant biomarker for HD patients. Hence, in this study, we explored BDNF levels and markers of BDNF gene activity in peripheral fluids, including plasma, whole blood and saliva in patients. With respect to plasma, we did not detect significant changes in plasma BDNF across diagnostic groups. Our results are consistent with previous findings showing no change in plasma or serum levels of BDNF in patients with HD (Zuccato et al., 2011). Although other studies have reported decreased levels of BDNF transcripts in whole blood of HD patients (Krzyszton-Russjan et al., 2013), and increased levels of BDNF protein in platelets from HD patients (Betti et al., 2018). Given the heterogeneous cell types found in whole blood, it is possible that BDNF levels could be altered in a cell-specific manner, which might explain these discrepancies. Other studies have shown that BDNF levels in plasma are different between females and males (Lommatzsch et al., 2005); however, we did not find significant sex differences in plasma BDNF in our cohort, regardless of disease status. We did detect significant positive correlations between both plasma and salivary BDNF and age, which is in contrast to previous studies in the literature that have found either no effect (Jiang et al., 2019) or modest decreased levels of BDNF with age in blood (Lommatzsch et al., 2005; Ziegenhorn et al., 2007). Importantly, however, we did not find that age was a significant covariate in analyzing the differences in BDNF levels between HD patients and controls.

In addition to plasma, we measured BDNF in saliva, which has been gaining recent attention as an alternative peripheral biofluid for biomarker studies (Kaufman and Lamster, 2002). Saliva has several advantages over blood, including its non-invasive nature, less sample processing and the ability to carry out sample collection in any setting, including at home. Here, we report a significant decrease of salivary BDNF in premanifest HD patients compared to control subjects, suggesting its potential application as an early marker of disease in pre-symptomatic mutation carriers. However, we did not detect any significant correlations between salivary or plasma BDNF levels with any clinical measure, including MMSE, TFC, HADS, or motor symptom scores.

We did not observe significant correlations between plasma and salivary BDNF levels, suggesting that salivary BDNF is not coming from the blood via known routes including transcellular transport, passive intracellular diffusion or active transport. BDNF in saliva could arise from expression in white blood cells, which are known to be present in saliva. Support for this idea comes from the fact that we did observe a significant negative correlation between salivary BDNF levels and whole blood DNA methylation at the *BDNF* promoter IV, although the *p*-value for this association did not survive multiple test correction, and white blood cells make up the majority of nucleated cells in whole blood. It is known that promoter methylation can directly regulate transcription (Razin and Cedar, 1991), hence, this correlation might provide a relevant basis for BDNF investigations in saliva. Additional studies using a larger cohort providing matched whole blood and saliva samples will be important to validate this finding. A further possibility is that BDNF is released into the saliva by the nerves innervating the salivary glands. This later possibility suggests that salivary BDNF may represent a better surrogate for brain tissue than plasma; a notion supported by previous observations of reduced BDNF protein levels in the brain of HD mouse models and human HD patients (Ferrer et al., 2000; Zuccato et al., 2001; Duan et al., 2003; Zhang et al., 2003; Corey-Bloom et al., 2017). Future studies should address the source of salivary BDNF, not only to understand fundamental mechanisms involved in the transportation of this protein, but also to better define the use of BDNF as a reliable disease biomarker for HD. Additionally, given that restoring BDNF levels may have therapeutic effects in HD (Zuccato et al., 2001; Gharami et al., 2008; Xie et al., 2010), BDNF measures might also represent a biomarker for monitoring novel therapeutics.

Studies have investigated DNA methylation alterations of the *BDNF* promoter, mainly forms I and IV, to serve as an epigenetic biomarker of disease states in association with neurodegenerative and neuropsychiatric pathology. In particular, changes in DNA methylation at BDNF promoters I and IV have been detected in Alzheimer’s disease (AD) (Rao et al., 2012; Xie et al., 2017), major depression (Januar et al., 2015; Kang et al., 2015), and schizophrenia (Ikegame et al., 2013), but such changes have not yet been explored in HD. Here, we report changes in DNA methylation at four CpG sites at promoter IV of the *BDNF* gene in HD patients compared to normal controls, with three site showing increased methylation and one site decreased in methylation. Interestingly, the three CpGs that showed increased methylation in HD cases are in close proximity, suggesting these sites may represent a functional unit that modulates BDNF transcription (Lovkvist et al., 2016).

Although chorea, consisting of involuntary and abnormal movements, is the most characteristic symptom of HD, this disease is also associated with cognitive and psychiatric manifestations that have a major impact on patients and family caregivers. Psychiatric symptoms including anxiety, social withdrawal, depression and impulsivity can often be present early in the disease and can significantly contribute to the overall disability (Sturrock and Leavitt, 2010). Notably, while BDNF promoter methylation was not correlated to motor and cognitive clinical symptoms, methylation at the three sites that showed increased levels in HD patients, was inversely correlated to HADS scores, which is a widely used self-report instrument for measuring anxiety and depression in somatically ill patients (Zigmond and Snaith, 1983; Brennan et al., 2010). This finding is highly relevant in light of previous studies showing associations between BDNF promoter methylation in psychiatric disorders, including schizophrenia and depression, as mentioned above, with one study specifically suggesting that BDNF promoter methylation could be used as a biomarker of depression (Januar et al., 2015). Although increased methylation at promoters usually represses transcription, BDNF is encoded by a highly complex gene harboring six promoters; hence it is likely that promoter regulation is complex and could involve other epigenetic regulators, such as histone modifications. Indeed, reduced levels of H3K4me3 and arginine methylation of H2A/H4R3 (mediated by PRMT5) at the BNDF promoter II have been reported in HD brains (Vashishtha et al., 2013; Ratovitski et al., 2015). Here again, although PRMT5-induced H4R2methylation is associated with gene silencing; chromatin immunoprecipitation experiments showed that this modification is also present in active genes; thus, PRMT5 may act as a positive regulator of BDNF as well; further illustrating the complexity of BDNF epigenetic regulation (Chen and Chen, 2017). Further studies are needed to unravel the epigenetic regulation of BDNF expression and may reveal a more intricate interplay between DNA methylation and salivary BDNF in association with depression symptoms.

DNA methylation is highly associated with aging, and the methylation status at particular genomic sites can be used as an epigenetic clock of aging. Methylation patterns are also distinctive in females and males; not only at the X and Y chromosomes but across autosomal chromosomes as well (Nishioka et al., 2012; Chan and Ye, 2017; Ihara et al., 2018). We tested for association of methylation at BDNF promoter IV with age and sex of the study participants. We did not find a significant correlation between DNA methylation and age, regardless of diagnosis. Although previous studies have reported sex differences in BDNF methylation potentially mediated by estrogen signaling (Chan and Ye, 2017), we did not observe significant differences in methylation between females and males, however, we did see trends toward increased levels methylation at certain loci in female controls. We cannot rule that the lack of association between age/sex and BDNF methylation at the promoter IV in our study is due to the small cohort size analyzed.

A limitation of our study is the use of DNA isolated from whole blood to quantify methylation. DNA methylation profiles are cell specific, therefore detection in blood may be confounded by the heterogeneous composition of the sample. Nonetheless, the mean methylation values we observed in our study, although small, are in good agreement with data reported for the same CpG sites in previous studies (Ikegame et al., 2013).

In summary, our findings demonstrate that salivary BDNF protein is lower in both PM and HD patients compared to controls. Within the PM group, we show that BDNF levels are significantly lower in those patients within 10 years to their predicted age to onset, suggesting the possibility that salivary BDNF could represent an early, non-invasive biomarker of disease onset. The increase in *BDNF* promoter methylation observed in the blood of HD patients and the correlation to psychiatric symptoms in HD may also be relevant for future biomarker utility.

## Data Availability Statement

The raw data supporting the conclusions of this manuscript will be made available by the authors, without undue reservation, to any qualified researcher.

## Ethics Statement

The studies involving human participants were reviewed and approved by the University of California, San Diego, Institutional Review Board. The patients/participants provided their written informed consent to participate in this study.

## Author Contributions

AG performed the experimental work, analyzed the data, and contributed to manuscript writing. JC-B provided blood and saliva samples, performed clinical evaluation of participants, and contributed to experimental design and manuscript preparation. ET performed the experimental work, analyzed the data, and contributed to experimental design and manuscript preparation. PD designed the methylation analysis, performed the data analysis, and contributed to overall experimental design and manuscript preparation.

## Conflict of Interest

The authors declare that the research was conducted in the absence of any commercial or financial relationships that could be construed as a potential conflict of interest.

## Footnotes

1 http://edwild.com/205-huntingtons-disease-conditional-onset-probability-calculator/

## Abbreviations

BDNF: brain-derived neurotrophic factor
HADS: Hospital Anxiety and Depression Scale
HD: Huntington’s disease
HTT: Huntingtin protein
MMSE: Mini-Mental State Examination
NA: not applicable
NC: normal control
PM: pre-manifest
TFC: Total Functional Capacity
UHDRS: Motor component of the Unified Huntington’s Disease Rating Scale.
