# Supplementary material for: Evaluation of Biochemical and Epigenetic Measures of Peripheral Brain-Derived Neurotrophic Factor (BDNF) as a Biomarker in Huntington’s Disease Patients
Source: Front Mol Neurosci. 2020 Jan 23;12:335. doi: 10.3389/fnmol.2019.00335 (PMC6989488; doi:10.3389/fnmol.2019.00335)
Supplement: Supplementary file 1 [file Data_Sheet_1.PDF]

Supplementary Figure 1.
Reproducibility of plasma BDNF measurements.

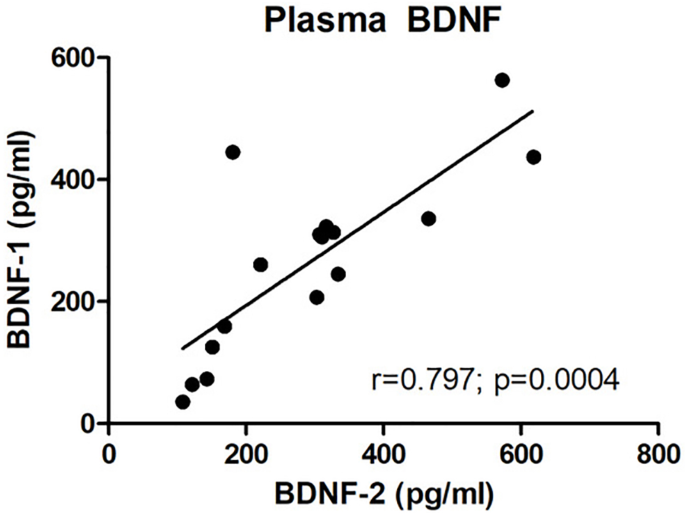

A subset of n=18 plasma samples (from all three cohorts) that we tested initially for BDNF protein measurements, were re-tested more than 1 year later using a similar BDNF ELISA. Data points showed good correlation with Pearson’s r=0.797 and p=0.0004. Data points represent individual BDNF values from subjects.
