# Supplementary material for: Evaluation of Biochemical and Epigenetic Measures of Peripheral Brain-Derived Neurotrophic Factor (BDNF) as a Biomarker in Huntington’s Disease Patients
Source: Front Mol Neurosci. 2020 Jan 23;12:335. doi: 10.3389/fnmol.2019.00335 (PMC6989488; doi:10.3389/fnmol.2019.00335)
Supplement: Supplementary file 2 [file Data_Sheet_2.PDF]

Supplementary Figure 2. Distribution of methylation at the BDNF promoter IV sites showing differential methylation in blood grouped according HD stage presentation.

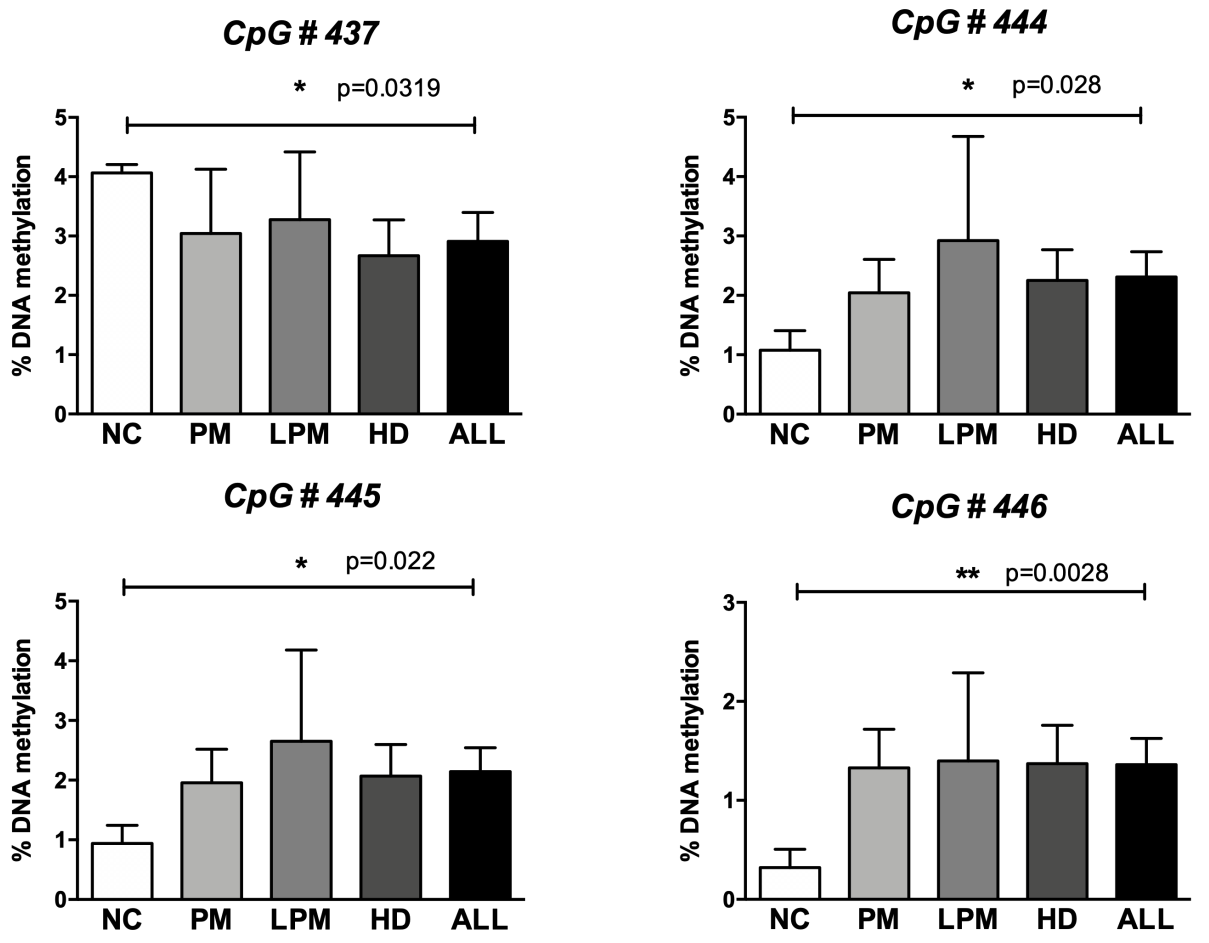

Methylation was quantified by pyrosequencing and expressed as mean percent methylation representing the averaged value for control subjects (NC); and cases (HD Gene +) for premanifest subjects (PM); late premanifest (LPM); symptomatic HD (HD); or combined cases PM + LPM + HD (ALL). of or for each of individual CpG. Data represents mean value ± S.E.M. *$p<0.05$ as per two-tailed Student's t-test.
