# Supplementary material for: Evaluation of Biochemical and Epigenetic Measures of Peripheral Brain-Derived Neurotrophic Factor (BDNF) as a Biomarker in Huntington’s Disease Patients
Source: Front Mol Neurosci. 2020 Jan 23;12:335. doi: 10.3389/fnmol.2019.00335 (PMC6989488; doi:10.3389/fnmol.2019.00335)
Supplement: Supplementary file 3 [file Data_Sheet_3.pdf]

Supplementary Figure 3. Distribution of DNA methylation at BDNF promoter IV by sex

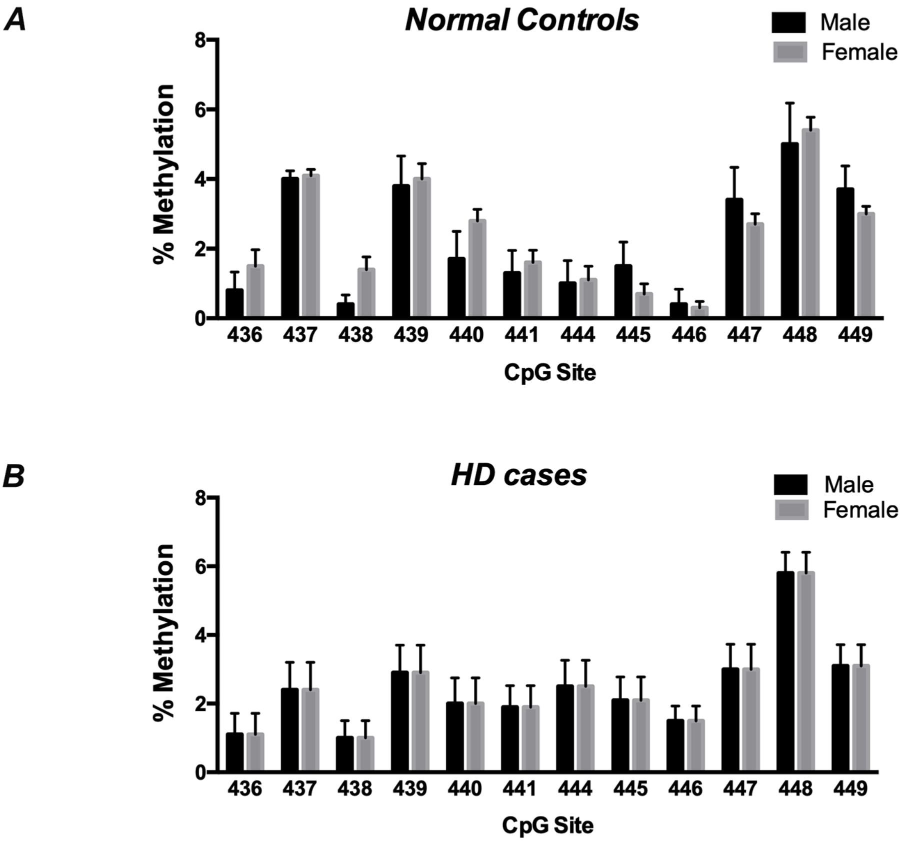

Plot of methylation levels at each individual CpG site tested at BDNF promoter IV grouped by sex in normal controls (A) and HD cases (B). Non sisgnificant differences were observed as per One-way ANOVA corrected by multiple observations.
